# Relationship between benthic macroinvertebrate bio-indices and physicochemical parameters of water: a tool for water resources managers

**DOI:** 10.1186/2052-336X-12-30

**Published:** 2014-01-10

**Authors:** Hamed Yazdian, Nematollah Jaafarzadeh, Banafsheh Zahraie

**Affiliations:** 1 School of Civil Engineering, College of Engineering, University of Tehran, Tehran, Iran; 2 Environmental Technology Research Center, Ahvaz Jondishapur University of Medical Science, Ahvaz, Iran; 3 Center of Excellence for Infrastructure Engineering and Management, College of Engineering, University of Tehran, Tehran, Iran

**Keywords:** Bio-diversity index, Physicochemical parameter, Genetic programming, Margalef index

## Abstract

The ecosystem health of rivers downstream of dams is among the issues that has become focus of attention of many researchers particularly in the recent years. This paper aims to deal with the question, how the environmental health of a river ecosystem can be addressed in water resources planning and management studies. In this study, different parameters affecting the ecosystem of river-reservoir systems, as well as various biological components of river ecosystems have been studied and among them, benthic macro-invertebrates have been selected. Among various bio-indices, biodiversity indices have been selected as the evaluation tool. The case study of this research is Aboulabbas River in Khuzestan province in Iran. The relationship between the biodiversity indices and physicochemical parameters have been studied using correlation analysis, Principal Component Analysis (PCA), and Genetic Programming (GP). Margalef index was selected as the appropriate bio-index for the studied catchment area. The relationship found in this study for the first time between the Margalef bio-index and physicochemical parameters of water in the Aboulabbas River has proved to be a useful tool for water resources managers to assess the ecosystem status when only physicochemical properties of water are known.

## Background

Bio-indices have been recognized as suitable criteria for understanding the quality of aquatic environment. They are numerical expressions that combine quantitative values of species diversity with qualitative information on the ecological sensitivity of each taxon [1]. Ecologists use various metrics and indices for ecological assessment of river ecosystem environments. They can be used to predict the response of an ecosystem to different water resources management practices and environmental conditions. Considering the importance of rivers, ecosystem environment and the role of bio-indices in basin scale water resources planning and management, most rivers in the developed countries are constantly evaluated and their physical, chemical and biological characteristics are monitored [2].

Various bio-indices have been proposed and used by ecologists in different countries. The most commonly used indices in biological evaluation of rivers include species richness, evenness, diversity and dominance indices, BMWP (Biological Monitoring Working Party), ASPT (Average Score per Taxon) and EPT (The total number of Ephemeroptera, Plecoptera and Trichopteraindex).

However the literature on the bio-indices and the criteria for understanding the quality of aquatic environment is rich, but there is a gap between these studies and those related to water resources planning and management. Most of the previous studies in the field of water resources planning and management have focused on socio-economic aspects of water allocation to different users while some also have considered physicochemical water quality constraints [3]. Bio-indices have not been used in these studies mostly because of the lack of knowledge of water resources modelers about these indices and also limited interval of limnological measurements. Previous studies some of which are also cited later in the section, show that the limnological information are only available in very short periods of time (mostly one or two years) in very limited rivers specially in the under developed countries while water resources planning and management studies require long records of data (usually longer than 30 years). To close this gap, one approach which is the focus of this study is to find a mathematical relationship between an ecological index which can reflect the overall environmental condition of a river in the study area and the physicochemical properties of water. Since there are widespread databases about physicochemical characteristics of water bodies in many basins around the world, finding this relationship can help in determining the quality of aquatic environments wherever no record on the quantity or diversity of species is available.

Several studies including the followings have shown consistency between variations of biotic indices and fluctuations in physicochemical characteristics of water: Czerniawska and Kusza [1], studied correlation between bio-indices and diversity indices at the family level of benthic macro-invertebrates with physicochemical variables of Nysa Klodzka River in southern Poland, using Spearman’s correlation coefficient.

Yap et al. [4] studied variations of a benthic species called Oligochateas and physicochemical parameters of water in a river in Malaysia from March 1998 to February 1999, and showed that there has been a negative correlation between density and distribution of this benthic macro-invertebrate and DO and PH, and a positive correlation with electrical conductivity, BOD, NO3, NH3, TSS, COD, Cc and Zn.

Azrina et al. [5] studied the correlation between richness and diversity index of benthic macro-invertebrates communities with physicochemical parameters of water of Langat River, Malaysia for four consecutive months (March–June 1999), and showed that they are mainly affected by TSS and EC of the river water. They showed that the richness index has a strong negative correlation with TSS, width of the river and temperature while Simpson diversity index is strongly correlated with TSS and electrical conductivity of water.

Latha and Thanga [6] in a study in India examined variations in Shannon diversity and evenness indices in a period of two years for six stations on the Veli and Kadinamkulam Rivers and showed that species diversity and distribution is clearly related to water quality and the more contaminated water is, the less the diversity index will be. Their study also showed that Shannon index has had fluctuations similar to abundance index.

Kennen et al. [7] studied benthic macro-invertebrates in 67 small and medium sized catchment areas in America and demonstrated the relationship between EPT species richness index and hydrological characteristics of flow.

In Iran, Nemati et al. [8] calculated various biotic indices estimated based on samples collected from benthic macro-invertebrates of Zayandeh-rud River. They studied correlation between these indices and physicochemical parameters of water and concluded that BMWP (Biological Monitoring Working Party) index has a significant correlation with physicochemical parameters of water.

Monk et al. [9] reviewed the 22-year long-term statistics of samples collected from 14 rivers in England. They computed BMWP, EPT and Life Score biotic indices and studied their variations with respect to changes in Indicators of Hydrologic Alteration (IHA) and observed the strongest relation between biotic indices and hydrological parameters in frequency and intensity of current flow groups.

Ogleni and Topal [10] studied the impacts of pollutants on water quality in 15 stations over Mudurnu River, Turkey in a 12-month period (2006 to 2007) and biotic indices obtained based on different organisms in water. They showed that from 100 biotic indices, 60% of them have used benthic macro-invertebrates and it seems that modified ASPT and BMWP indices have the strongest correlation with water quality parameters.

The above studies show that different types of bio-indices have statistically significant relationships with hydrological indicators of flow and physicochemical characteristics of water. All of the aforementioned studies have used descriptive statistics to assess this relationship. However, These types of assessments could be useful for many environmental planning and management purposes, but they cannot be used for inclusion in the operation management models of river-reservoir systems. The questions this study is trying to answer are: 1) When modeling river-reservoir systems, which bio-index should be chosen? And 2) How the relationship between the chosen bio-index and physicochemical characteristics of water can be quantified?

The case study of this research is Aboulabbas River in Khuzestan Province in Iran. Genetic Programming (GP) has been used in this study to obtain a quantitative relationship between biodiversity index and physicochemical characteristics of water.

## Materials and methods

### Using benthic macro-invertebrates for calculating bio-index

Different biotic indices have been defined and used in different regions of the world for bio-monitoring programs, which some of them have a reasonable accuracy to be used in other regions too. Biological assessments can be used for identifying weaknesses in ecosystem environments caused by pollutants or degradation of habitats. They are also, in some cases, even more effective than physical and chemical measurement processes, because they are economical and need less time to be evaluated.

Among the various components of the aquatic ecosystems including plants, birds, fish and Macrobenthic organisms (Macrobenthos), the last one pave the way for one of the best and most efficient ways for biological assessments [11]. Macrobenthos plays the role of a link in food chains which provide the energy stored by plants in larger animals such as fish. Aquatic invertebrates in the river food chains are the primary consumers of herbal products such as algae, diatoms, mosses and decaying leaves and enter the production cycle of the fish, and when mature, they fly or they are directly consumed by secondary consumers.

Macrobenthos are invertebrates which can be seen with the naked eye. They spend at least part of their lives in the river beds. Being the basic components of the aquatic chains of rivers and ubiquitous in all aquatic ecosystems, limited mobility, long lifespan and species richness with varying sensitivity to pollution are the highlighted reasons for widely reported studies on benthic macroinvertebrares as biological monitoring techniques [5,12-14].

Exploiting benthic macro-invertebrates is based on the assumption that the streams and rivers which are not affected by pollutant factors have more arrays of benthic species and non-resistant species are dominant there, while in polluted waters, arrays which are less tolerant to pollutants can be found less [13].

### Parameters affecting the ecosystem of rivers

The first step for choosing an appropriate bio-index and obtaining its possible mathematical relationship with physicochemical characteristics of river water is identifying the parameters with considerable effects on the ecosystem of the river being studied. Studying the mathematical relationship between variations of biotic indices with these physicochemical characteristics is the second step. In this step, the proper biotic index which shows strongest statistical relation with the physicochemical parameters can be selected. Some of the most influencing physicochemical characteristics of the river water bodies on the ecosystems can be listed as follows:

•River discharge is the most important hydrologic characteristic of rivers. It has direct and indirect impacts on the ecosystem health. While river discharge directly satisfies the needs of species in rivers, indirectly change the physical and chemical quality of water.

•Water velocity is among the major characteristics affecting river ecosystems. It has significant effects on morphology of river beds and movement of sediments which both have impacts on various species Floods and all types of hydrologic alterations can significantly change the ecosystem health one way or another.

•In addition to the hydrological conditions of the river, water quality parameters also play a major role in ecosystem health. Any change in water quality can lead to variations in compositions of plants and animal species. The most important water quality parameters in terms of impact on aquatic ecosystems include temperature, salinity, acidity, Total Dissolved Solids (TDS), pH, DO and BOD_5_. Many physical processes and chemical and biological transformations are sensitive to temperature variations. Salinity increase in freshwater ecosystems generally decreases biodiversity and may reduce the available food resources. Generally lower acidity leads to reduced biodiversity and species composition of various invertebrate communities. Increased turbidity reduces light penetration depth and thus limits the growth of aquatic species. Since oxygen is needed for aerobic respiration of aquatic species, low DO concentration is harmful to plants and aquatic organisms [15-17].

### Bio-indices

Various bio-indices have been proposed and used by ecologists in different countries, such as species richness index, evenness index, species diversity index, dominance index, and BMWP, EPT and ASPT indices.

Evenness index demonstrate the distribution of the communities of species. The more even species distribution is, (i.e. the number of individual organisms or abundance of species are more similar), the higher stability is present which results in greater biodiversity. Species richness indicates the presence of various species and is calculated by the number of species in an area. An increasing number of taxons can be due to habitat diversity, suitability of water or its improved quality. Dominance index reflects the abundance of some species over others which is used as an index in biodiversity assessments. Species diversity index is in fact a combination of species richness and evenness indices, and aggregate both species richness and evenness into a single quantity. Higher biodiversity indices indicate less stress in ecosystems, higher abundance and more even distribution of species in the ecosystem. Various studies have also shown this point, some of which are also cited in this paper.

With respect to the various biotic indices, it seems that using diversity indices for river ecosystem health assessment will be more appropriate [18-20], stated that diversity index increases by increased number of species or increasing the total number of organisms in populations; when the population of various species is distributed evenly, the diversity index increases as well.

Shannon, Simpson, and Margalef diversity indices have been used by several researchers to assess bio diversity. These indices have been also used in this study and therefor are introduced with more details in the following sections.

### Shannon diversity index

Shannon diversity index has been a popular diversity index in the ecological literature. It was originally proposed by Claude Shannon in 1948. This index can be estimated using the following formula after estimating the relative abundance of identified families at each station for different months of a year:

(1) $$\text{SHI}=\sum_{i=1}^{s}P_{i\times }\ln P_{i}$$

Where:

*P*_*i*_: Relative abundance of *i*^th^ taxon in the sample.

*s*: total number of taxons in the sample.

It has been emphasized in the literature that Shannon diversity index is a fast and reliable tool to identify major changes in community structure of benthic species [21]. It has also been shown that seasonal patterns of Shannon diversity index and species richness and evenness are similar to seasonal changes in species abundance and composition [22].

### Simpson diversity index

Simpson diversity index was presented by Simpson in 1949. In 1972, Krabs presented the following formula for estimating Simpson diversity index:

(2) $$\mathrm{SI}=1-\sum_{i=1}^{s}{P_{i}}^{2}$$

In this index, lower/higher weights are assigned to the rare/usual species. The index values are in the range of zero (lowest diversity) to $\left(1-\frac{1}{S}\right)$ (highest diversity).

### Margalef diversity index

In 1958, Margalef introduced this index as a simple diversity index.

(3) $$\mathrm{MI}=\frac{\left(s-1\right)}{\ln N}$$

Where *N* is the total number of individuals.

In order to find the relationship between bio-indices and the physicochemical characteristics of river, GP has been used in this study. This technique is briefly described in the following section

### Genetic algorithm for programming (Genetic programming)

To obtain a formula indicating the relationship between biotic index and qualitative and quantitative characteristics of water, GP has been used. GP was proposed for the first time by Koza in 1992 [23]. The first step in GP is generating initial population randomly consisting of two elements, i.e. functions and terminals. Functions can, according to the type of problem, be the basic operations like addition, subtraction, multiplication and division or logical functions such as AND, OR and NOT or any other function. Terminals also include variables and constants, if desired.

In GP, functions and terminals are randomly selected, and a member of population is presented as a tree with functions as its roots and branches that ultimately end to the terminals. After generating a random initial population which is known as the parent for the first generation, each member will be evaluated and this evaluation can be carried out in different ways based on the type of problem. From initial population, a new population is formed using various selection methods such as roulette wheel, tournament, etc. GP operators including “reproduction,” “cross over” and “mutation”, affect this new population [24].

GP has proved to be a useful tool especially when the relationship between variables is unknown or the size and form of relationship is complex and difficult to formulate, as well as when no approach can be presented by analytical and mathematical methods for establishing relationship between variables [25,26].

In application of GP for determining the relationship between bio-indices and physicochemical characteristics of water, firstly, all parameters have been standardized to be in the range of [0, 1] to avoid any magnitude difference between the parameters. The basic mathematical operators of addition, subtraction, multiplication, and division have been considered as functions. GP offers a different relation for calculating bio-index in each run. Due to the fact that GP, like other evolutionary methods, is based on producing initial random answers, the estimated equation in each run can be different. Various relationships between the dependent variable (biotic index) and the independent variables (qualitative and quantitative parameters) are calculated using the results of 100 runs of GP. The best relationship is then selected based on the highest correlation coefficient. It worth mentioning that since GP algorithm uses random operators, it is suggested in the literature that the final results should be chosen from several runs.

To formulate a relationship between a bio-diversity index and physicochemical parameters of water, by removing discharge variable from independent variables, again GP is used. 80% of the available dataset has been used for training and 20% for validation. The GP parameter used in this study are as follows:

•mutation rate = 0.1;

•population size = 300;

•maximum number of generations = 500;

•Functional set = addition, subtraction, multiplication and division.

### Case study

In Iran, very few studies on aquatic ecosystems can be found and there is very little information available. In the recent years, some efforts have been spent to further recognize and assess aquatic environments in some catchment areas.

**Figure 1:**
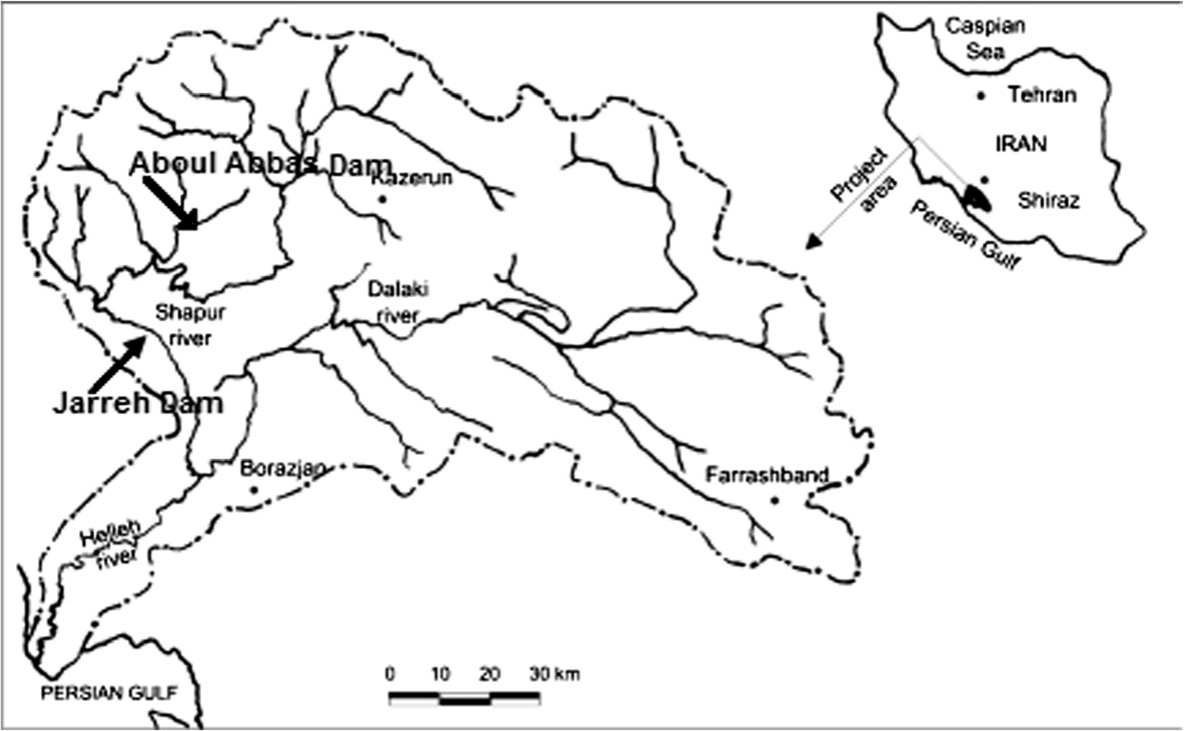
Location of Aboulabbas river and reservoir.

The case study of this research is Aboulabbas River located in the southwest of Iran in Khuzestan Province, between 25˚ 31́′ to 31˚ 40′ North latitudes and 50˚ 49′ to 50˚ 10′ East longitudes (Figure 1). Samplings have been carried out in six stations around Aboulabbas dam in the period of January 2007 to December 2007. Available samples include number of fish and benthic macro-invertebrates and also physicochemical parameters of water on a monthly basis.

**Figure 2:**
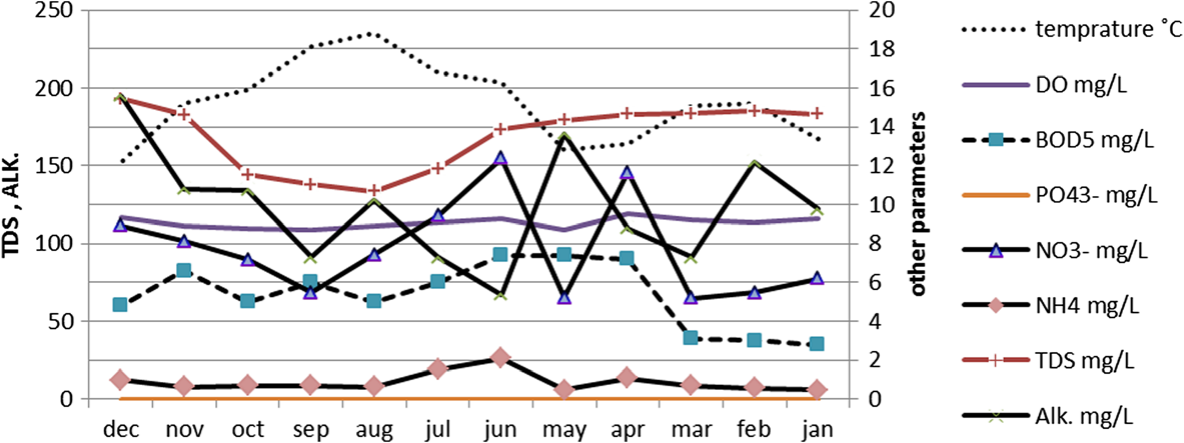
Physicochemical parameters of the Aboulabbas river water in the period of January 2007 to December 2007.

As shown in Figure 2, the available data shows that generally the river water quality is good. For example, dissolved oxygen in all cases was reported to be more than 7.6 mg/L which place the river in Class 1A according to the national water quality standards of Iran. The maximum amount of measured dissolved solids was 200 mg/L while this amount should not be greater than 500 mg/L as recommended by EPA for drinking water. BOD_5_ is also in a range which is suitable for irrigation (Figure 2). There is no significant source of industrial or chemical pollution in the catchment basin of this river. Therefore, it is assumed that biotic indices are affected only by natural conditions of the river and are not affected by the pollutants.

It worth mentioning that no sampling has been carried out after 2007. Since no major development or land use change has happened in the basin, it is assumed that the results of this study are still valid for water resources planning purposes.

## Results and discussions

In order to establish a relationship between bio-indices and physicochemical parameters of water, firstly, based on the existing information of the catchment area, Simpson, Shannon and Margalef diversity indices have been calculated for 12 months of the year at different stations. Then using SPSS software program, the correlation coefficient between biotic indices with quantitative parameters (river discharge) and qualitative parameters (Water Temperature, pH, DO, EC, BOD_5_) have been calculated. Analysis of the results revealed a significant correlation between bio-indices and relatively high correlations between bio-indices and some of the physicochemical parameters (Table 1).

**Table 1:** Correlation between bio-indices and physicochemical parameters of the Aboulabbas river data

**Correlation**	**Tem.**	**Q**	**pH**	**DO**	**EC**	**BOD**	**TDS**	**Shannon**	**Simpson**	**Margalef**
Temperature	1.000	−.244	−.114	−.789	.594	.124	.592	−.336	−.232	−.501
Q		1.000	−.020	.254	−.214	−.014	−.215	.020	.034	.078
pH			1.000	−.249	−.337	.349	−.335	−.055	−.115	.068
DO				1.000	−.205	−.339	−.203	.338	.301	.368
EC					1.000	−.175	1.000	−.153	−.076	−.350
BOD						1.000	−.174	−.074	−.063	−.097
TDS							1.000	−.154	−.078	−.351
Shannon.								1.000	.967	.850
Simpson.									1.000	.749
Margalef.										1.000

For conducting a more accurate analysis, the available data for all of the stations, including biotic indices and qualitative and quantitative parameters have been clustered using K-means clustering technique. K-means clustering is a simple clustering method with low computational complexity. It is very simple and can be easily implemented in solving many practical problems. K-means algorithm is under the category of Squared Error-Based Clustering [27]. For all of three selected bio-indices, it has been observed that the data for winter season has been clustered into one cluster and the data for the rest of the year in another cluster. Bearing this point in mind for further analysis, and in order to establish a relationship between bio-indices and physicochemical parameters, the data which is clustered into one cluster and has the information related to spring, summer and autumn is used in GP. Since TDS and EC parameters are highly correlated, only EC has been used as independent variable.

Hundred GP runs provided equations for estimating each of the biotic indices with various degrees of accuracy. The obtained results presented in the Table 2 show the number of presence of each of the physicochemical parameters in the obtained equations for calculating each of the biotic indices. The results show that a small percentage of the obtained equations for calculating all of the bio-indices are affected by the river discharge. Moreover, DO and BOD_5_ parameters have the most frequent repetition in the obtained equations.

One of the questions that should be answered here is which of the physicochemical parameters should be included in the estimation of bio-indices. As it can be seen in Table 2, different combinations of physicochemical parameters have been used in GP for estimating bio-indices. In order to answer this question, Principal Component Analysis (PCA) method has been used. PCA is a multivariate statistical analysis technique, which has been widely used in the water quality related studies [28-31]. The results of PCA are shown in Table 3. The results of PCA show that PC1-PC4 factors contain more than 80 percent of information and by reviewing Table 3 it can be concluded that DO and temperature parameters are more important for the first main component, EC parameter for the second main component, pH for the third component, and BOD_5_ parameter for the fourth component. The PCA results also show low importance of discharge compared with other parameters investigated in estimating bio-indices. Therefore, the PCA results are compatible with the GP outcomes.

**Table 2:** Percent of presence of each physicochemical parameter in the equations obtained from GP for calculating the bio-indices

**Bio-indices**	**Physicochemical parameter**					
	**Q**	**Temperature**	**DO**	**BOD**_**5**_	**pH**	**EC**
Shannon	10%	32%	62%	60%	50%	23%
Simpson	14%	43%	39%	81%	29%	32%
Margalef	19%	44%	63%	55%	49%	67%

**Table 3:** Rotated component matrix

	**Component**			
	**PC1**	**PC2**	**PC3**	**PC4**
Temperature	.861	.401	−.109	.049
Q	−.131	−.092	−.013	.000
pH	.062	−.170	.969	.169
DO	−.947	−.040	−.168	−.177
EC	.216	.949	−.179	−.099
BOD_5_	.150	−.086	.166	.971

**Table 4:** Comparison of statistics (mean, standard deviation and correlation coefficient) between observed and calculated values of the Margalef index

**Margalef index**	**Training**			**Validation**		
	**Observed**	**Calculated**	**Estimation error (%)**	**Observed**	**Calculated**	**Estimation error (%)**
Mean	1.33	1.28	3.8	1.19	1.13	5.04
Std. deviation	0.45	0.48	6.6	0.43	0.48	11.6
*R*^2^	0.632			0.738		
Mean square error	0.159			0.104		

Correlation analysis has been carried out between the estimated values of the three bio-indices based on the observations and the estimated values using the equations obtained from GP. As it was mentioned earlier, 100 values have been estimated for each index. The results of the correlation analysis shows that the estimated values by GP method for Margalef diversity index has higher correlation with the values estimated based on the observations. Therefore, Margalef biotic index is chosen in this study.

The equations generated in GP have been evaluated by two goodness-of-fit measures, root-mean-square error and correlation coefficicent. Based on these two measures, Equation (1) had the highest fitness based on both measures:

(4) $$\mathrm{MI}=\frac{\mathrm{DO}}{\left(T+2*\mathrm{DO}\right)\left(T+\mathrm{EC}+\mathrm{BOD}5\right)}$$

Where:

*MI*: Margalef diversity index,

*DO:* dissolved oxygen (mg L^-1^),

*T:* water temperature (°C),

*EC:* Electrical Conductivity of the water (μmohs cm^-1^), and

*BOD*_5_: Biological oxygen demand (mg L^-1^).

To assess the accuracy of this relationship, summary statistics of the observed and estimated values of the index are presented in Table 4.

Reviewing the results reveals the relatively significant accuracy of the obtained relationship in both training and validation datasets. Table 4 indicates that the error of the equation in estimating the average value of Margalef index is about 3.8% and 5.04% for the training and validation datasets, respectively. There has been 6.6% and 11.60% difference between standard deviation of the calculated and observed values of the index in training and validation datasets, respectively. The correlation coefficients between the observed and estimated values of the Margalef index estimated for training and testing datasets show relatively acceptable accuracy of the proposed relationship. Mean square error for training and validation datasets have been relatively close which shows no over fitting has occurred.

**Figure 3:**
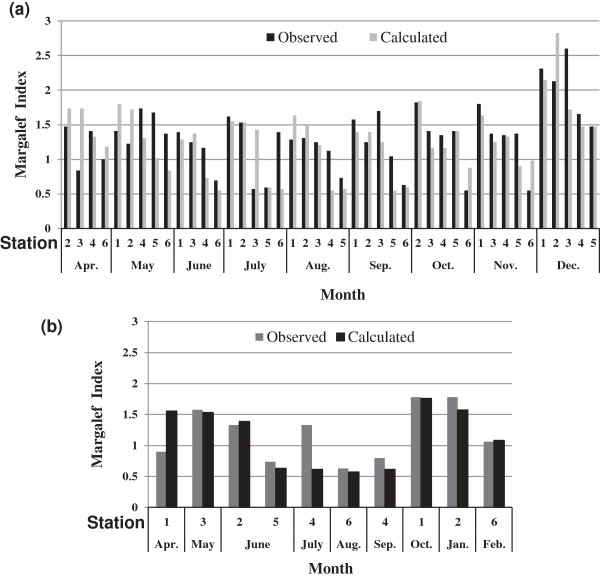
**Comparison between calculated and observed values of margalef index in the period of January 2007 to December 2007 at different stations. (a)** training. **(b)** validation.

Figure 3 shows comparison between the calculated and observed Margalef diversity index at different stations on Aboulabbas River. As it can be seen in this figure, the highest error of estimations by Equation 4 compared with observations has been in the month of April. It is worth mentioning that the river discharge in April is the highest in the year, and such surplus discharge often in the form of flash floods causes sudden changes in the river ecosystem. Excluding the results obtained for the month of April increases the correlation coefficients and this implies that the obtained formula is more accurate for other months of the year. Due to reduced river discharge and increased temperatures and reduced water quality in summer and autumn, the health of ecosystem is usually at stake in these months, so maintaining ecosystem health and improving biodiversity in such months is more important for water resources planners, and this equation can be a useful tool for calculating biotic indices in these months whenever there is no measurement.

## Conclusion

The aim of this study has been to provide a tool for assessing biodiversity of river ecosystems to be used by water resources planners and reservoir operators. The major obstacle in this study has been the lack of long-term data. The accuracy of the proposed equation can be significantly improved in case of availability of long-term observations. Despite this fact, the novelty of this work lies in the methodology used in choosing biotic indices and the physiochemical parameters for estimating them. The equation proposed in this study for estimating Margalef index is based on the environmental condition of the study region and we are not to claim that it would work in other regions as well as Aboulabbas River, because diversity and even abundance of benthic macroinvertebrates depend on various physico-chemical properties of water and specific environmental condition of each ecosystem. Also, a larger dataset could lead to more accurate mathematical relationships between ecological target indices and various water quality parameters.

Further research can be dedicated to finding similar equations for other rivers in the region specially the headwaters of Aboulabbas River to assess whether the same conclusion about the choice of bio index and physicochemical parameters is valid for them. For the larger datasets, it can also be suggested to investigate the possibility of increasing the accuracy of the relationship by making it sensitive to the overall pollution level of the river water.

## Competing interests

The authors declare that they have no competing interests.

## Authors’ contributions

HY carried out this research under the guidance of NJ and BZ. All authors read and approved the final manuscript.
